# Olfaction in People with Down Syndrome: A Comprehensive Assessment across Four Decades of Age

**DOI:** 10.1371/journal.pone.0146486

**Published:** 2016-01-05

**Authors:** Maria Paola Cecchini, Dario Viviani, Marco Sandri, Antje Hähner, Thomas Hummel, Carlo Zancanaro

**Affiliations:** 1 Department of Neurological and Movement Sciences, Anatomy and Histology Section, University of Verona, Verona, Italy; 2 U.O. Pediatria, Ospedale Sacro Cuore Don Calabria, Negrar, Italy; 3 Smell & Taste Clinic, Department of Otorhinolaryngology, University of Dresden Medical School, Dresden, Germany; University of Graz, AUSTRIA

## Abstract

**Background:**

Down syndrome (DS) shows neuropathology similar to Alzheimer disease, which presents olfactory impairment. Previous work showed olfactory impairment in DS, but a comprehensive evaluation of olfactory function in DS is lacking.

**Methods:**

We investigated a large number (n = 56; M = 31, F = 25) DS participants (age range18-57y) using the “Sniffin’ Sticks” Extended test. This comprises three subtests (threshold, discrimination, and identification) yielding a global score (TDI) defining normosmia, hyposmia, and functional anosmia. To the best of our knowledge, this is the second largest group of DS people investigated for olfactory function ever. Age- and sex matched euploid individuals (n = 53) were the control.

**Results:**

In DS, TDI was lower (16.7±5.13 vs. 35.4±3.74; P<0.001), with DS people performing worse in any subtests (P<0.001 for all); 27 DS participants showed functional anosmia (i.e., TDI<16). In DS, age was weakly and negatively correlated with TDI (r = -0.28, P = 0.036) and identification (r = -0.34, P = 0.012). When participants were stratified in young adults (18-29y) and older adults (30-61y), a significant effect of age was found for identification in both DS (young adults, 8.3±2.58; older adults, 6.9±2.99; P = 0.031) and control (young-adult, 14.3±1.18, older adult, 13.0±1.54; P = 0.016).

**Conclusion:**

Olfactory function is overall severely impaired in DS people and may be globally impaired at relatively young age, despite of reportedly normal smell. However, specificity of this olfactory profile to DS should be considered with some caution because cognition was not evaluated in all DS participants and comparison with a control group of non-DS individuals having cognitive disabilities was lacking. Further study is required to longitudinally assess olfactory dysfunction in DS and to correlate it with brain pathology.

## Background

In the brain of individuals affected with Down syndrome (DS), a trisomy 21 disorder, neurodegenerative changes take place, which are similar to those found in Alzheimer disease (AD) namely, neuritic plaques, neurofibrillary tangles, amyloid deposits, cerebral atrophy, and loss of presynaptic cholinergic markers [1–5]. In DS, deposition of amyloid in the form of senile plaques or diffuse amyloid deposit has been found in cortical brain regions associated with olfactory processing (e.g., entorhinal cortex) as early as the age of 19y [6–8]. Accordingly, olfactory deficits have been described in AD [9] and DS since many years [10–13]; however, nasal function in DS has been found to be comparable to controls [14] suggesting that olfactory deficits are secondary to central-nervous rather than rhinological pathology.

Given the dramatic increase in prevalence and distribution of plaques by the third and fourth decade of life in DS [15] and the progressive brain disease found in DS (similar to AD [2]) it has been hypothesized that aging negatively affects the olfactory deficit in DS. This may represent a serious issue for DS people, given their increase in longevity. In fact, early in this century mean survival was 61.1y for males and 57.8y for females born with DS in Australia [16] with the calculated mean age of death for children with DS being approximately 9y in 1933 [17]; at present, there may be over 210,000 people with DS over the age of 55 in the United States of America [18]. Nevertheless, in a recent comprehensive review on aging in DS [18] the olfactory deficit was not considered, suggesting that research is needed to explore the pattern of olfactory function across the whole life span in DS. Actually, the number of studies investigating olfactory function in DS is quite limited [12, 13, 14, 19, 20, 21, 22, 23, 24]; one third of these papers [13, 20, 23] investigated to some extent the role of aging in the olfactory deficit of DS. Moreover, most studies are focused on individual aspects of olfactory function thereby lacking a comprehensive evaluation.

In the clinical setting, the complexity of olfactory function may be assessed with non-invasive psychophysical tests exploring performance in specific olfactory tasks (e.g., odor detection, discrimination, identification, memory, suprathreshold intensity). Importantly, these different olfactory tests seem to tap into different functions of the olfactory system [25, 26]. Accordingly, in this study we jointly measured olfactory threshold, olfactory discrimination, and olfactory identification performance in each participant by means of a standardized test battery.

The test yields a global score, the TDI score (= threshold score + discrimination score + identification score) allowing for detection of normosmia (TDI≥30.3), hyposmia (30.3>TDI≥16), and functional anosmia (TDI<16). Kobal et al. introduced the term “functional anosmia” in 2000. This definition means that subjects with a TDI score below 16 are considered to be completely anosmic or to have some olfactory function left, which is not useful in daily life [27]. Indeed, a subject with functional anosmia may still perceive few odors, discriminate between some of them or show olfactory event-related potentials [28]; however, this residual olfactory function does not contribute to the enjoyment of food/drink or to the detection of spoiled food or gas leaks. The above procedure was validated on more than 3000 healthy subjects [27] and it is widely used in Europe but, to our knowledge, it has never been used in DS individuals.

In the current work, such a comprehensive evaluation of olfactory function was carried out in a large sample of DS people and age-matched euploid controls over an age range of four decades with a two-fold aim: first, to investigate olfactory function in DS; second, to study the possible age-related impairment of olfactory function in DS. Given the above mentioned increase in prevalence and distribution of amyloid plaques by the third and fourth decade of life in the DS brain [15] the effect of age on olfactory tests performance was assessed by stratifying the sample into two age groups: young-adult (age range, 18-29y) and older-adult (age range, 30-61y). The effect of sex (M, F) was assessed as well.

## Methods

### Participants

DS individuals were recruited through public and private organizations working in the field of mental disability. Inclusion criteria were age ≥18y, overall good health, ability to comprehend and perform adequately the test procedure. Exclusion criteria were presence of pathology able to affect olfactory performance (e.g., recent head trauma, rhinitis or chronic sinus infection, diabetes, stroke, thyroid dysfunction, history of smoking/alcohol). The study protocol and the olfactory test procedures were illustrated to DS individuals and/or guardians, and written informed consent obtained. Control subjects were recruited through public advertisement. All investigations were carried out in accordance with the Helsinki Declaration and the protocol was approved by the relevant IRB at the University of Verona (Prot. N. 102, 3^rd^ April 2012, TIT.11/9).

Prior to olfactory testing all participants were asked to rate their olfactory function and the answer (better than normal, normal, less than normal) was registered.

### Procedures

Olfactory testing was carried out in a well-ventilated room using the “Sniffin’ Sticks” Extended test (Burghart, Wedel, Germany). This is a test of nasal chemosensory performance based on pen-like odor dispensing devices with a felt-tip (length 14 cm, diameter 1.3 cm). Instead of liquid dye, the pen's tampon is filled with 4 ml of a given liquid odorant or odorant dissolved in propylene glycol. The test consists of three subtests namely, threshold (the concentration at which the odor is reliably detected), discrimination (the ability of the subject to distinguish odors) and identification (the subject has to identify 16 different odors from a list of 4 odors each). In order to increase the reliability of the measurements, each subject must give an answer (forced choice paradigm). During each subtest, the experimenter removes the cap of the pens and the pen's tip is held approximately 1 cm under both nostrils for around 3 seconds. Patients are tested blindfolded with a sleeping mask to prevent visual identification of the odorant-containing pens for threshold and discrimination test.

The threshold subtest uses n-butanol as target odor (ink pen odor). Threshold is assessed using a staircased, three alternative forced choice procedure (3-AFC procedure). It consists of sixteen dilutions, prepared in a geometric series starting from a 4% n-butanol solution (dilution ratio 1:2 in deionized water as solvent). Three pens are presented in randomized order, with two containing the solvent and one containing the odorant. Subjects are asked to identify the odor-containing pen. Reversal of the staircase (i.e. the presentation of the triplet with the next lower odor concentration) is started when the odor-containing pen is correctly identified in two successive trials. When subjects give an incorrect answer, the triplet with the next higher odor concentration is presented and thus, the staircase is reversed. Testing is completed after seven reversals of the staircase. Odor threshold is defined as the mean of the last four out of seven staircase reversals.

The discrimination subtest also uses a 3-AFC procedure: triplets of pens are presented in randomized order, with two containing the same and one a different odorant. Subjects have to determine which of the three pens smells different than the other two pens. For threshold and discrimination subtest the presentation of triplets is separated by 30 seconds. The interval between administrations of individual pens is approximately 3 seconds.

The identification subtest is assessed for 16 common odors (e.g., orange, peppermint, coffee, fish, banana). Immediately before the odor is presented, the examiner reads a list of four response options from which the subject has to choose the correct answer. The subject is asked to choose one answer option that s/he feels to be correct after the odor had been presented, having also the possibility to read a paper words list and look at pictures of the four choice options each time, before and after the odor presentation [29, 30]. The interval between odor presentations is approximately 30 seconds. The decision to add to the paper words list odor’s pictures was adopted to give a support in words’ meaning comprehension to the DS subjects [31]. This olfactory task with pictures seems to be particularly appropriate for children and for individuals with mental disability as reported in [23]. We used pictures taken from various sources [freely available on www.google.it/] because a validated olfactory identification test with pictures corresponding to the Burghart identification odors did not exist.

Overall, 60 persons with DS were recruited. Four participants were discarded due to a family consent refusal *a priori* or because of going-on difficulties arisen during the procedure. Data from a final group of 56 DS participants (31 M; 25 F), age range 18-57y were used for analysis. A group of euploid participants (n = 53; M = 23, F = 30, age range: 18-61y) fulfilling the same inclusion and exclusion criteria were used as control.

For a subset of DS participants (n = 13; 5M, 8F, mean age: 26.4±4.19y, age range: 18-33y) cognitive evaluation was available. An expert psychologist tested cognition with the Wechsler Adult Intelligence Scale-Revised (WAIS-R). This scale consists of six verbal and five performance subtests. The verbal tests are: Information, Comprehension, Arithmetic, Digit Span, Similarities, and Vocabulary. The Performance subtests are: Picture Arrangement, Picture Completion, Block Design, Object Assembly, and Digit Symbol. Thus, a Verbal Intelligence Quotient (VIQ), Performance Intelligence Quotient (PIQ), and full scale IQ were available (TIQ).

### Data analysis

Normal distribution of the data was assessed using the Shapiro-Wilk test. The following variables were approximately normally distributed: discrimination, identification, and TDI. Threshold, VIQ, PIQ, and TIQ variables had a non-normal distribution; accordingly, nonparametric statistical methods were adopted in the analysis of these variables. Numerical variables were summarized using mean, standard deviations (SD) or median, Inter-Quartile Range (IQR) as appropriate. Student t test and chi-square (χ^2^) test were used to compare mean age and the proportion of females in the DS and control group, respectively.

Three-way ANOVA was used to investigate the differences in discrimination, identification, and TDI between the 8 groups identified by three binary factors: DS/control, M/F, and age class (young adult, age range 18-29y; older adult, age range 30-61y). The same analysis was performed for threshold using median regression with interactions terms (and sum-to-zero coding for factors). Eta-squared (η^2^) estimates of effect size were reported for the main effects of each factor, together with F statistics (t statistics for threshold) and P-values. The mean differences in olfactory tests by sex and age were also investigated in control and DS separately using linear regression (median regression for threshold). Wald test on linear and median regression coefficients was performed to evaluate the significance of these differences (t statistic is reported with P-value).

Correlation was assessed using the Pearson’s correlation coefficient (r) or the Spearman’s rank correlation coefficient (ρ) where appropriate. Nonparametric estimates of the area under the ROC curve (AUC-ROC) were calculated to assess the ability of olfactory tests in discriminating DS from controls.

Differences were considered significant at P≤0.05. Statistical analyses were performed using Stata 13 (StataCorp, College Station, TX).

## Results

### Characteristics of the participants

The demographic data for the participants are reported in Table 1 together with the relevant statistics.

**Table 1:** Demographic characteristics of participants. Means are ± SD.

Characteristic	Down syndrome	Control	Test on Difference^§^
	(n = 56)	(n = 53)	
Sex			χ^2^ (1,N = 109) = 1.56; P = 0.212
M	31 (55.4%)	23 (43.4%)	
F	25 (44.6%)	30 (56.6%)	
Age (y)	30.1±10.74	28.3±11.08	t(107) = -0.86; P = 0.394

^§^ = chi-square test for Sex and Student’s t-test for Age

The DS and control groups were not significantly different for age and sex. All participants reported to have a normal sense of smell. The experimenter administering the olfactory test noted no difficulties with sniffing performance in any subjects. No signals of fatigue in the course of the experiment were noticed. None of the participants was taking drugs that are known to have an influence on olfactory perception. The TDI score was above the threshold for normosmia (i.e., 30) in all but one euploid participant. Instead, 27 DS individuals (20M, 7F) out of 56 showed functional anosmia (i.e., TDI<16).

### Differences in olfactory tests performance—DS/controls

**Fig 1:**
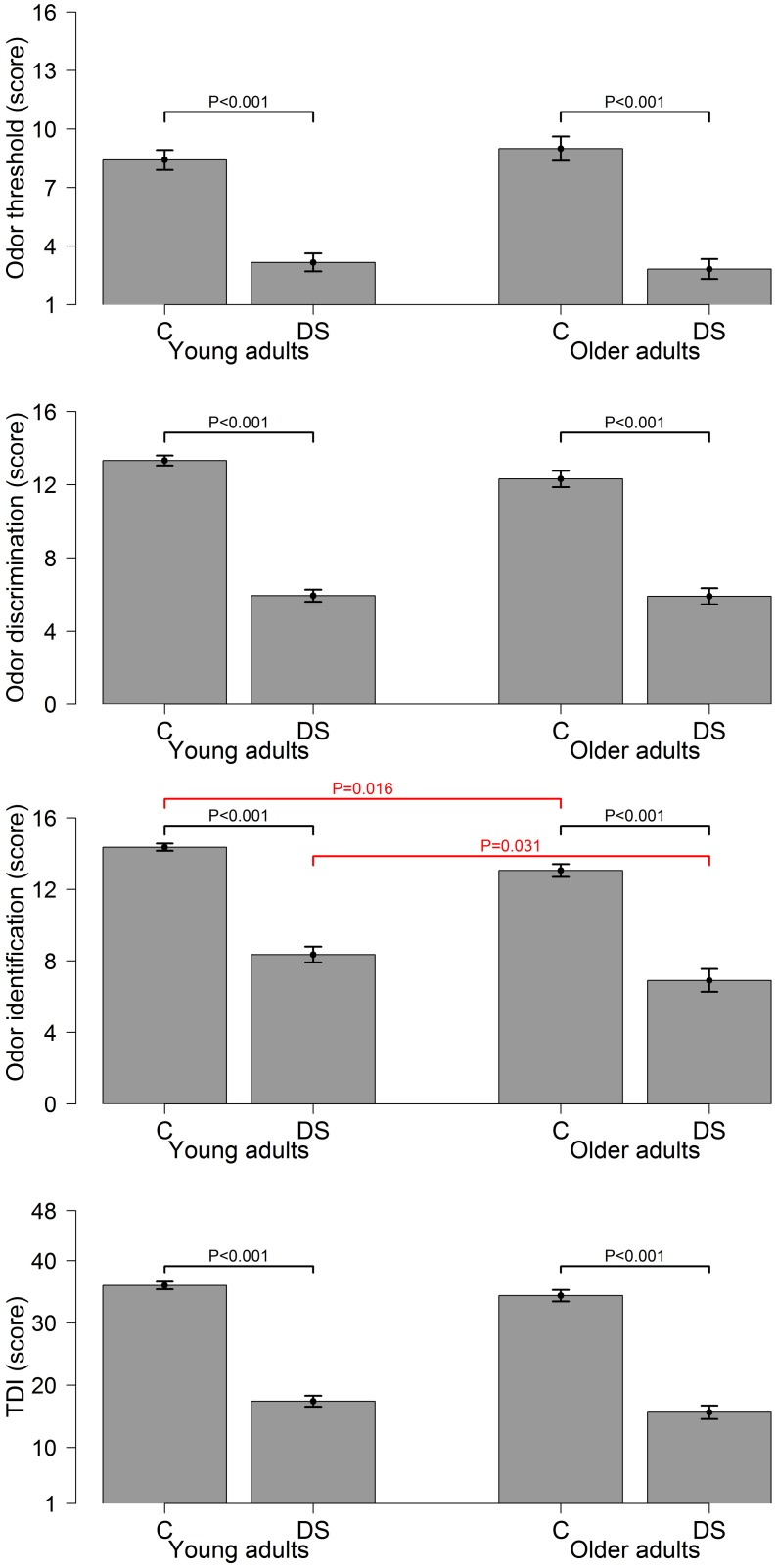
Mean scores (with 95% confidence intervals) estimated for DS individuals (DS) and euploid controls (C) in each olfactory subtest (threshold, discrimination, identification), and the global test (TDI) according to age class (young adult [<30y], left panel; older adult [≥30y], right panel). Olfactory performance (adjusted by sex) was significantly lower in DS than controls for any tasks and TDI in both age classes (P<0.001 for all, black bars). Identification score was significantly higher in young adults than older adults both for controls (P = 0.016) and DS (P = 0.031, red bars).

ANOVA showed that mean TDI score was 16.7±5.13 (range 5.0–27.5) in DS and 35.4±3.75 (range 27.2–46.0) in control group (F(1,101) = 422.49;P < 0.001; η^2^ = 0.81). The DS group performed significantly worse than controls in any subtests: threshold, 2.1 (IQR = 1.0–4.1) vs. 8.3 (IQR = 6.5–9.7) (t(101) = -10.18; P < 0.001; η^2^ = 0.53); identification, 7.8±2.81 vs. 13.9±1.45 (F(1,101) = 179.11;P < 0.001; η^2^ = 0.64); discrimination, 5.9±1.97 vs. 13.0±1.77 (F(1,101) = 343.84; P<0.001; η^2^ = 0.77). Using linear regression models, further analysis was conducted in each age class (young adult, older adult) adjusting by sex. A significant difference was found for any subtests and for TDI with P<0.001 for all: identification in young adults (DS = 8.4±2.58 vs. control = 14.4±1.18; t(65) = -12.18) and in older adults (DS = 6.9±2.99 vs. control = 13.1±1.54; t(38) = -7.97); threshold in young adults [DS = 2.3 (IQR 1.0–4.3) vs. control = 8.1 (IQR 6.5–9.5); t(65) = -7.20) and in older adults [DS = 1.9 (IQR 1.0–3.5) vs. control = 8.5 (IQR 6.8–10.5); t(38) = -6.24]; discrimination in young adults (DS = 5.9±1.92 vs. control = 13.3±1.59; t(65) = -17.07) and in older adults (DS = 5.9±2.09 vs. control = 12.3±1.95; t(38) = -10.15); TDI in young adults (DS = 17.4±5.16 vs. control = 36.0±3.52; t(65) = -17.42) and in older adults (DS = 15.6±5.01 vs. control = 34.4±3.99; t(38) = -13.36). Results are shown in Fig 1. The ability of olfactory tests in discriminating DS from control was excellent (AUC-ROC>0.90 for all). TDI showed the highest predictivity; for a threshold value TDI<27.5, overall accuracy, sensitivity and specificity were 99.1%, 100%, and 98.1%, respectively.

### Differences in olfactory tests performance—Age

ANOVA showed a significant main effect of age for odor identification in the whole cohort (young adult 11.4±3.62; older adult 9.8±3.92; F(1,101) = 6.78, P = 0.011; η^2^ = 0.06). Non-significant differences were found for threshold [5.9 (IQR = 1.9–8.6) vs. 5.8 (IQR = 1.5–8.5); t(101) = 0.90, P = 0.368; η^2^ = 0.10], discrimination (9.6±4.11 vs. 8.9±3.80; F(1,101) = 1.34; P = 0.250;η^2^ = 0.01), and TDI (26.7±10.33 vs. 24.3±10.47, F(1,101) = 1.52, P = 0.212; η^2^ = 0.01). Using linear regression models, further analysis was conducted in each group (DS, control) by age class (<30y, ≥30y) adjusting by sex. Results are shown in Fig 1. A significant effect of age class was found for identification in both the DS (young adult, 8.3±2.58; older adult, 6.9±2.99; t(53) = -2.21, P = 0.031) and control (young adult, 14.3±1.18, older adult, 13.0±1.54; t(50) = -2.49, P = 0.016) groups.

### Differences in olfactory tests performance—Sex

When sex (M, F) was considered in the whole cohort, a significant main effect was found for TDI (M = 23.3±10.43, F = 28.2±9.86; F(1,101) = 9.44, P = 0.003; η^2^ = 0.09) and threshold (M = 3.5 [IQR = 1.3–7.5], F = 6.8 [IQR = 3.8–9.5], η^2^ = 0.06; t(101) = 3.39, P = 0.001), with difference in discrimination (M = 8.6±4.18, F = 10.0±3.71,; F(1,101) = 3.50, P = 0.064; η^2^ = 0.03) and identification (M = 10.0±3.91, F = 11.5±3.55; F(1,101) = 3.65, P = 0.059; η^2^ = 0.03) being at the limit of statistical significance. The role of sex was also investigated in the control and DS group separately by regression analysis, adjusting by the effect of age. A significant difference was found for identification (M = 13.5±1.50, F = 14.2±1.35; t(50) = 2.58, P = 0.013) in the control group, as well as TDI (M = 15.2±4.94, F = 18.6±4.80; t(53) = 2.52, P = 0.015), threshold (M = 2.3±2.07, F = 3.9±2.85; t(53) = 3.08, P = 0.003), and discrimination (M = 5.5±2.09, F = 6.4±1.73; t (53) = 3.42, P = 0.001) in the DS group.

### Correlational analysis

In the whole cohort (n = 109), age weakly and negatively correlated with the scores of all olfactory tests; statistical significance was found for TDI (r (107) = -0.19, P = 0.044) and identification (r (107) = -0.24, P = 0.012); after adjusting for sex, odor identification only retained significance (r (106) = -0.20, P = 0.038). In the DS group (n = 56), age showed weakly significant negative correlation with TDI (r (54) = -0.28, P = 0.036) and identification (r (54) = -0.34, P = 0.012); correlation with threshold (ρ (54) = -0.11, P = 0.431) and discrimination (r (54) = -0.04, P = 0.784) was not significant. After adjusting for sex, identification only retained significance (r (53) = -0.30, P = 0.026). In the control sample (n = 53), similar result was found for TDI (r (51) = -0.28, P = 0.042) and threshold (ρ(51) = -0.02, P = 0.898); the correlation between age and discrimination was weakly significant (r (51) = -0.27, P = 0.050) and borderline significant for identification (r (51) = -0.25, P = 0.066). After adjusting for sex, no correlation retained significance.

In the subset of DS patients undergoing the WAIS-R test, weak to moderate positive correlations were found between scores in olfactory and cognitive tests (Table 2). In particular, statistically significant correlations were found between discrimination, identification and TDI, and VIQ and full IQ.

**Table 2:** Pairwise correlations (threshold: Spearman’s ρ; discrimination, identification, and TDI: Pearson’s r) between olfactory and cognitive tests in DS individuals (n = 13).

	WAIS-R		
Olfactory test	VIQ	PIQ	TIQ
Threshold	0.36	0.09	0.21
Discrimination	0.66*	0.33	0.57*
Identification	0.57*	0.42	0.64*
TDI	0.78**	0.40	0.68*

VIQ, Verbal Intelligence Quotient; PIQ, Performance Intelligence Quotient; TIQ, full scale Intelligence Quotient; TDI, sum of threshold, discrimination, and identification scores

*, P <0.05;

**, P <0.01

## Discussion

This work investigated in some detail the olfactory function in the second largest sample of people with DS ever. Data were obtained across about four decades of age, and compared with those from age- and sex-matched euploid individuals. Results demonstrate the following points:

1. The overall olfactory function (odor threshold, odor discrimination, odor identification) is severely impaired in both young-adult (<30y) and older-adult (>30y) DS individuals.
2. Aging is associated with limited loss of olfactory function, especially odor identification.

A first finding of this study is that olfactory threshold, olfactory discrimination and olfactory identification are all severely impaired in DS. Since all DS subjects reported to have a normal sense of smell, it is apparent that they are not aware of their olfactory *status*. Therefore, it is important that DS subjects and caregivers are educated to appreciate the relevance of olfaction in daily life, especially in recognizing dangerous odours (e.g. gas, smoke or spoiled food). However, similar to what is known from patients with disorders leading to a gradual smell loss (e.g. Alzheimer’s or Parkinson’s disease [32]), there is also a large number of healthy adults with a slowly progressive olfactory dysfunction unaware of their deficit [33] and, in consequence, the presence of olfactory dysfunction is often underestimated in the general population [34, 35]. Accordingly, DS *per se* may be not the only determinant of the lack of awareness of smell deficit in DS participants. In this study, the relative contribution of DS status and the progressive olfactory dysfunction found in the general population was not assessed.

The current results are supported by previous work showing, in nine adult (average age 25.9y) DS individuals, a 44% lower performance in the University of Pennsylvania Smell Identification Test (UPSIT) [36] vs. matched controls [19]. Similarly, UPSIT performance was 47% lower in twenty younger DS persons/individuals (mean age 20.0±2.3y) vs. euploid matched controls [13]. In a study involving 23 DS individuals (30.1±7.2y, range 25-46y) Murphy and Jinich (1996) found a 47% lower performance in UPSIT vs. euploid matched controls; moreover, using n-butyl alcohol as the stimulus, Murphy and Jinich (1996) showed that mean odour detection threshold was sharply lower in DS than control (dilution step 5.0 vs. 8.6). In the largest study to date, involving 67 DS individuals (age range from 7–50 years), Nijar and Murphy (2002) investigated odor threshold (n-butyl alcohol dilution test) and odor identification (San Diego Odor Identification Test, SDOIT [37] and UPSIT) in DS vs. normal individuals and mentally retarded subjects. Nijar and Murphy (2002) found generally poorer performance in DS at the child (7-17y), young-adult (18-29y), and adult (30-50y) age level. For example, odor threshold was 44% and 46% lower in young-adult and adult DS individuals vs. normal controls. In SDOIT, the mean percentage of correctly identified odors was 62.5% and 96% in the DS and normal control group, respectively; for UPSIT, performance was markedly worse in DS vs. normal controls at the three age levels. In the present study, we used the validated [27] “Sniffin’ Sticks” Extended Test exploring odor threshold, odor discrimination, and odor identification to confirm and expand on previous results by showing that the DS group generally performed worse than euploid controls (threshold, -65%; discrimination, -55%; identification, -44%; TDI, -53%).

A novel finding of the present work is that olfactory discrimination is impaired in DS. To our knowledge, olfactory discrimination has never been assessed in DS but in a single study [21]. In this study, 20 young DS adolescents (average age 13 years) were evaluated with a 16 items three choice test, scratch and sniff method, and findings showed lower mean performance in DS adolescents vs. age-matched controls, but the difference was not statistically significant. While the limited number of participants in this study may have prevented statistical significance, the reported absence of AD related pathology in early teens might play a role, supporting the speculation by McKeown and colleagues that DS related olfactory dysfunction occurs only at ages when AD-like pathology is present and/or for an initial period of time brain compensatory mechanisms operate preserving olfactory function. In our study, the minimum age for inclusion was 18y, thereby preventing assessment of olfactory function in younger DS individuals; however, we found that in DS young adults (18-29y) olfactory discrimination is impaired (as well as olfactory threshold and olfactory identification), suggesting that olfaction in DS is overall impaired at relatively young age. Further work involving e.g. structural and functional magnetic resonance imaging is required to more precisely correlate olfactory dysfunction and brain pathology in DS.

The evidence for age-related deficits in olfaction is overwhelming [27] (for a recent review see [38]), the cause being manifold and involving both neural and non-neural factors. In DS, an increased risk of several chronic diseases that are typically associated with older age is present. This may be due to the ability of trisomy 21 to accelerate aging, for example by increasing the biological age of tissues [39].

Earlier work carried out in 20 DS individuals (including 9 older subjects with mean age 32.2±7.10y) showed a trend for increased olfactory identification deficit with age [13]. While the small number of participants may have affected significance, these findings could indicate that the age effect on olfactory deficit is fully expressed at age >30y. In this work we examined a sample of DS individuals including about 40% of individuals aged 30+y and found moderate, negative correlation between age and olfactory performance as well as a significant effect of age class (<30y, ≥30y) for identification. Similar results were found in age-matched controls. Accordingly, it is suggested that the age-associated olfactory deficit roughly parallels in DS and euploid individuals, with odor identification being mainly affected by age. These findings are supported by previous data showing poorer performance of 14 older DS persons (mean age 41±6.8y, range 32-54y) vs. younger DS subjects aged 26.5±3.0y (range 20-31y) in an olfactory naming task [20]; in a larger number of adult (n = 26, mean age 38.8±7.1y) and young-adult (n = 23, mean age 23.2±4.0) DS individuals Nijjar and Murphy (2002) showed a non-significant trend for reduced odor threshold in the former and a significant reduction of picture-based odor identification.

Although not within the main focus of this investigation, a further result of this work is that females with DS have less impaired olfactory function than males over a large age range. The question as to whether olfactory performance is to some extent sex-related, similar to perceptual motor or verbal tasks, has been debated for decades. Investigation of sex difference in olfactory threshold (i.e., olfactory sensitivity) yielded an overall evidence for females being more sensitive than males for some odorants (reviewed in [40]). The evidence in favour of females’ better performance in odor identification is more consistent, with several studies showing superior female performance in identifying every day’s life common odors as well as human body odors [40]. Using the “Sniffin’ Sticks” Extended test, Hummel et al. (2007) found that, in 3282 male and female subjects, female outperformed males in the three olfactory tests. In this work we showed that DS females have significantly higher TDI score (an estimate of overall olfactory function) than DS males, suggesting that the neurobiological basis for better olfactory function in females is not affected by trisomy 21.

A first limitation of this work is that cognition was not evaluated in all DS participants. Accordingly, the effect of mental retardation on olfactory performance could not be evaluated in full. A second limitation of our work is the lack of comparison with another control group with non-DS individuals having cognitive disabilities. This would support the specificity of this olfactory profile to DS.

Assessment of olfactory threshold, discrimination and identification allows a measure of the integrity of the olfactory function. In spite of this, it is important to mention that also the personal cognitive profile has an influence on higher order olfactory performance [41,42]. Odor detection does contribute to the odor-identification deficit, but does not account for it completely [43] indicating that odor threshold taps into a different olfactory domain compared to odor identification and odor discrimination (see also [1,2]).

However, WAIS-R measurements positively correlated with tests of olfactory performance in the subsample where this information was available (Table 2), in agreement with previous work showing significant positive correlation between UPSIT scores and Dementia rating Scale in DS [12]. In particular, the statistically significant correlation we found between higher order olfactory performances and VQI, even if in a small sample of subjects, supports previous studies showing that there is a positive relation between proficiency in semantic tasks with an important verbal component, and both discrimination and identification tasks [25, 42, 44]. Interestingly, the strength of correlation was definitely lower for threshold than identification, discrimination, and TDI. An explanation for this finding is that in threshold test, although a 3-AFC paradigm is used, the pen containing the odor may be easily identified. In contrast, during odor discrimination testing, the subject has to memorize the smell of the other two pens before completing the task. Memory of odors is even more so required during odor identification test [26]. During the odor identification process, detection, discrimination, recognition and retrieval of an odor name are involved [9, 25, 45, 46]. Moreover, it is important to mention that previous studies revealed that DS individuals have relatively poor explicit memory ability when compared to control subjects, even using olfactory stimuli (see [47]). So, the influence of memory might be a significant factor to be considered in DS people. Therefore, odor discrimination and odor identification appear to involve higher-level cognitive functions compared to odor thresholds, and it may be hypothesized that the reported effects relate to the interaction between cognitive and olfactory function. Accordingly, odor identification and discrimination appear to be not only an indicator for olfactory function but also for cognitive abilities.

It is interesting to note that the amyloid precursor protein (APP) gene is located on chromosome 21. This gene encodes the transmembrane protein APP, from which Amyloid β is produced. The extra copy of APP gene is believed to lead to overproduction of Amyloid β peptides in DS [48]. This appears to be critical for the development of AD. Thus, the features of AD in DS individuals could be present 20–30 years earlier. Accordingly to the above, this “accelerated degeneration process” in DS could explain for example the global olfactory tasks deficit. Evidence suggests that in early phases of AD, olfactory impairment is addressed on more cognitively driven tasks (i.e. discrimination and identification), while odor threshold is spared [49]. In DS also odor threshold is severely impaired, even in people under 30y. Since odor threshold has been shown to correlate with the olfactory bulb volume [50], and olfactory bulb is the first central relay station of the peripheral olfactory neurons, it is possible that in DS the fast degeneration process leads to a severe deficit even at a relatively young age also on olfactory tasks of lower cognitive order such as odor threshold. However, in the absence of neuropathological data relating neurodegeneration and olfactory function in DS, the above considerations remain speculative.

## Conclusion

The current study investigated olfactory function over four decades of age in a large adult population with DS assessing olfactory threshold, olfactory identification and, for the first time, olfactory discrimination. It was apparent that, despite of reportedly normal smell, olfactory function is overall severely impaired in DS people group, such an impairment appearing at relatively young age. Accordingly, DS people and caregivers should better appreciate the relevance of olfaction in recognizing e.g., dangerous odors. In a subsample of participants, olfactory performance correlated with cognitive performance, indicating interaction between cognitive and olfactory function. Further studies are required both to compare olfactory and cognitive performances also in a group of non-DS individuals with cognitive disabilities and to further investigate olfactory system by e.g., nasal brushing and magnetic resonance imaging in order to correlate clinical findings with brain pathology insofar the olfactory deficit may represent an early indicator of neurodegenerative events occurring in the DS brain.
